# Cathepsin K analysis in a pycnodysostosis cohort: demographic, genotypic and phenotypic features

**DOI:** 10.1186/1750-1172-9-60

**Published:** 2014-04-26

**Authors:** Ahmet Arman, Abdullah Bereket, Ajda Coker, Pelin Özlem Şimşek Kiper, Tülay Güran, Behzat Özkan, Zeynep Atay, Teoman Akçay, Belma Haliloglu, Koray Boduroglu, Yasemin Alanay, Serap Turan

**Affiliations:** 1 The Department of Medical Genetics, Marmara University, Istanbul, Turkey; 2 The Department of Pediatric Endocrinology, Marmara University, İstanbul, Turkey; 3 The Department of Pediatric Genetics, Hacettepe University, Ankara, Turkey; 4 Department of Pediatric Endocrinology, Medeniyet University, Istanbul, Turkey; 5 The Department of Molecular Biology and Genetics, Istanbul Kultur University, Istanbul, Turkey; 6 Pediatric Genetics, Department of Pediatrics, Acibadem University School of Medicine, İstanbul, Turkey

**Keywords:** Cathepsin K, Pycnodysostosis, Fracture, Craniosynostosis, Arnold Chiari malformation

## Abstract

**Background:**

To characterize *cathepsin K (CTSK)* mutations in a group of patients with pycnodysostosis, who presented with either short stature or atypical fractures to pediatric endocrinology or dysmorphic features to pediatric genetics clinics.

**Methods:**

Seven exons and exon/intron boundaries of *CTSK* gene for the children and their families were amplified with PCR and sequenced. Sixteen patients from 14 families with pycnodysostosis, presenting with typical dysmorphic features, short stature, frequent fractures and osteosclerosis, were included in the study.

**Results:**

We identified five missense mutations (M1I, I249T, L7P, D80Y and D169N), one nonsense mutation (R312X) and one 301 bp insertion in intron 7, which is revealed as Alu sequence; among them, only L7P and I249 were described previously. The mutations were homozygous in all cases, and the families mostly originated from the region where consanguineous marriage rate is the highest. Patients with M1I mutation had fractures, at younger ages than the other pycnodysostosis cases in our cohort which were most probably related to the severity of mutation, since M1I initiates the translation, and mutation might lead to the complete absence of the protein. The typical finding of pycnodysostosis, acroosteolysis, could not be detected in two patients, although other patients carrying the same mutations had acroosteolysis. Additionally, none of the previously described hot spot mutations were seen in our cohort; indeed, L7P and R312X were the most frequently detected mutations.

**Conclusions:**

We described a large cohort of pycnodysostosis patients with genetic and phenotypic features, and, first Alu sequence insertion in pycnodysostosis.

## Background

Pycnodysostosis is a rare autosomal recessive disorder characterized by short stature, acroosteolysis of the distal phalanges, clavicular dysplasia, osteosclerosis with increased bone fragility, and delayed closure of sutures [1-6]. To date, less than 200 cases with equal sex distribution have been reported worldwide and, estimated prevalence is 1 to 1.7 per million [6]. The gene locus responsible for the Pycnodysostosis had been mapped to human chromosome 1q21 by genetic linkage analysis [7,8] and the gene encoding *cathepsin K* (*CTSK*) was identified through the positional cloning strategy [9]. Cathepsin K is a lysosomal cysteine protease which is a member of the papain-cysteine protease family involved in the degradation of bone matrix proteins, type I and type II collagen, osteopontin, and osteonectin at low pH [10,11]. The *CTSK* genomic DNA spans 12 kb and contains 8 exons and 7 introns. The translation initiation codon methionine (Met1) is located in exon 2, whereas the termination codon is located in exon 8. cDNA of *CTSK* encodes 329 amino acid protein including 15-amino acid signal peptide encoded by part of exon 2, a 99 amino acid proregion encoded by the parts of exon 2, exon 3, and the part of exon 4 and, 215-amino acid mature active enzyme encoded by the parts of exon 4, exon 5, 6, 7, and 8 [12].

CTSK is synthesized as an inactive precursor protein, and requires removal of its N-terminal proregion for activation [13]. This autocatalytic process occurs under a low-pH environment [14,15].

At least thirty-four different mutations have been identified in the *CTSK* gene and these mutations are nonsense, missense, frameshift, splice site mutations, small deletions and insertions. Majority of the mutations are located at the mature active domain of CTSK protein [6].

In this study, the coding region and exon/intron boundaries of the *CTSK* gene were analyzed for mutations in sixteen children from fourteen families with Pycnodysostosis. Five missense mutations (M1I, I249T, L7P, D80Y and D169N), one nonsense (R312X ) and one insertion mutation were identified. Five of the described mutations are novel (M1I, D80Y, D169N, R312X and insertion mutations).

## Methods

### Patients

**Figure 1:**
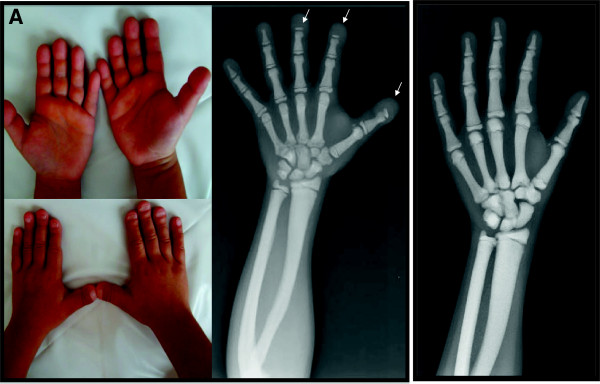
**Panel A: Typical finding of acro-osteolytic distal phalanges on X-rays and wrinkled skin over the dorsa of distal fingers and flattened and grooved nails in pycnodysostosis detected in patient 4.** Panel **B**: Osteosclerosis without acroosteolysis detected on radiograph of the patient #8.

Sixteen patients from 14 families were studied. Patients #1-I and #1-II are distant cousins, while patients #13-II.1 and #13-II.3 are the sisters. Nine patients were evaluated in the Pediatric Endocrinology Clinics in Istanbul and Erzurum due to short stature, while seven patients were referred to a Pediatric Genetic Unit in Ankara for dysmorphic features. The diagnosis was based on their phenotypic and radiographic evaluation. The clinical features of the patients are summarized on Table 1, in which the number of fractures and height standard deviation scores (SDS) are representing data at the time of presentation. Figure 1 shows typical acroosteolysis and osteosclerosis, which are the most prominent radiological and morphological feature of the disease. However, patients #8 and #13.II did not show any acroosteolysis, osteosclerosis and typical facial feature leading to diagnosis. As an additional finding, one of the patients had Arnold Chiari malformation (Patient #6) and patient #8 had craniosynostosis.

**Table 1:** Clinical and demographic features of the patients with pycnodysostosis summarized according to mutations

**Pt ID**	**City of Origin**	**Centers**	**Age (F/M)**	**Open Ant. Fontanel**	**Acroosteolysis**	Fractures (n)- age (yr)*	**Height SDS**
M1I-(3G- > A)- Exon 2							
1-I	Ordu	Istanbul	2.7 (F)	Yes	Yes	Cranium (1)-2.3	-3.01
1-II			6.5 (F)	No	Yes	Femur (2)-5.4	-2.36
L7P- (20 T - > C)-Exon2							
2	Ankara	Ankara	32 (M)	No	Yes	Femur (2), tibia (1)	-4.3
3	Ankara	Ankara	10 (M)	Yes	Yes	Tibia (1)	-3.9
4	Çorum	Istanbul	11 (F)	No	Yes	Tibia (1)-9.7	-4.8
5	Samsun	Ankara	19 (M)	Yes	Yes	Tarsal (1)-14	-3.8
D80Y-(238G- > T)-Exon 3							
6	Kastamonu	Istanbul	10.5 (F)	No	Yes	Yes**-10.5	-2.31
D169N-(505G- > A)-Exon 5							
7	Erzincan	Erzurum	5 (M)	Yes	Yes	No	-2.2
I249T-(746 T- > C)-Exon 6							
8	Sivas	Istanbul	6 (F)	No	No	No	-2.6
9	Yozgat	Ankara	12 (M)	Yes	Yes	Tibia (3), clavicle (1), scapula (1)-6	-3.6
R312X-(934C- > T)- Exon 8							
10	Yozgat	Ankara	14 (M)	Yes	Yes	No	-4.4
11	Corum	Ankara	8 (F)	Yes	Yes	No	-5.2
12	Erzincan	İstanbul	9.8 (M)	No	Yes	Tibia (3)-5	-2.12
N296fX54 (IVS7-14-15insAlu: HSU18392)-Intron 7							
13-II.1	Mardin	Istanbul	6.5 (F)	Yes	Yes	No	-4.64
13-II.3			1 (F)	Yes	No	No	-1.77
14	Batman	Ankara	16 m (F)	Yes	Yes	No	-2.0

*The age given for fractures is the age the first fracture occurred in the patient. **Cervical vertebrate during operation for Arnold Chiari malformation.

Marmara University ethics committee approved the study and, written informed consents were obtained from the patients and parents for the genetic studies.

### Molecular genetic analysis

#### *DNA isolation and specific exon and exon/intron boundary PCR*

Genomic DNAs were isolated from blood samples of the patients and their families according to salting out method [16], and kept at 4°C. The exons 2–4 and 6–7 together, and exon 5 and 8 separately and their flanking splice sites were amplified by polymerase chain reaction (PCR) using the primers shown in Additional file 1: Table S1.

PCR reactions were performed in presence of 500 ng genomic DNA, 1X PCR buffer, 1U taq polymerase, 200 μM each deoxynucleotide triphosphate, 0.25 μM each primer and, cycling conditions for exons 2-4, 5, 6-7 and 8 were (94°C, 3 min)x1; 94°C, 30 sec, 54°C, 30 sec, 72°C, 45 sec)X35; 72°C, 10 min)X1. PCR products were visualized on 2% agarose gels to rule out large deletion and insertions.

### DNA sequencing

The amplified PCR products for *CTSK* gene were purified and sequenced with direct sequencing of the DNA Cycle sequencing System (ABI Prism kit) with the dideoxy-chain termination method and applied on an autosequencer (ABI Prism 377 DNA sequencer).

#### *Data analysis*

Sequencing traces were analyzed through BLAST database (NCBI) and Web Map Preferences (Harvard) and, acceptor splice site in the insertion sequence was determined by Human Splicing Finder-Version 2.4.1.

## Results

Seven exons and exon/intron boundaries of *CTSK* gene for the children and their families were amplified with PCR and sequenced. Five different missense mutations were determined in the Cathepsin K gene. Patients #1-I and #1-II were cousins and showed 3 G > A mutation located at exon 2 and the G residue of translation initiation codon ATG was converted to A (ATA). This mutation is homozygous (Additional file 2: Figure S1A) and is also novel. This mutation changes the translation initiation codon and leads to no protein synthesis (M1I), since there is no in-frame Kozak sequence in the following exons and, significantly reduced protein synthesis in L7P mutation, which is located in the signaling peptide sequence, has been shown [4].

The L7P homozygous mutation was observed in patients #2, #3, #4 and #5, and this mutation was reported previously in an Italian family [4]. L7P mutation occurred by changing of the T residue of CTG codon to C residue resulting to CCG at exon 2 (Additional file 2: Figure S1B). This mutation was previously shown to affect CTSK protein targeting due to disruption in signal peptide sequence of protein [4].

The D80Y mutation was located at exon 3 of the *CTSK* gene and observed in patient #6. This mutation is homozygous and occurs by changing of the G residue of GAC codon encoding D to T, creating the TAC encoding Y (Additional file 2: Figure S1C, shown in reverse sequence C to A). This mutation is also novel and was found at the prodomain of CTSK. This mutation changed highly charged hydrophilic D to less hydrophobic bulky Y amino acid; this probably affects protein folding of CTSK protein.

Patient #7 showed D169N mutation and this mutation is located at exon 5. This mutation occurs by changing of the G residue of GAT codon encoding D to A to form AAT encoding N. This mutation is also a novel mutation (Additional file 2: Figure S1D and Additional file 3: Figure S2). This mutation changed highly hydrophilic, negatively charged, D residue to uncharged amino acid N of CTSK, and this change might be detrimental to protein folding.

Another homozygous missense mutation I249T was located at exon 6 in patients #8 and #9 and, this mutation was created by changing of the first T residue of ATT encoding I to C to form ACT encoding T amino acid (Additional file 2: Figure S1E and Additional file 3: Figure S2). This mutation was previously reported and affects the hydrophobic cluster of CTSK protein resulting in unfolding structure of the protein [4].

R312X mutation was observed in patients #10, #11 and #12, and this mutation is homozygous and novel (Additional file 2: Figure S1F). This mutation was caused by changing of the C residue of CGA encoding R to T resulted in TGA, stop codon on *CTSK* gene. Patients carrying this mutation will have a CTSK missing 17 amino acid residues from the carboxy terminal.

**Figure 2:**
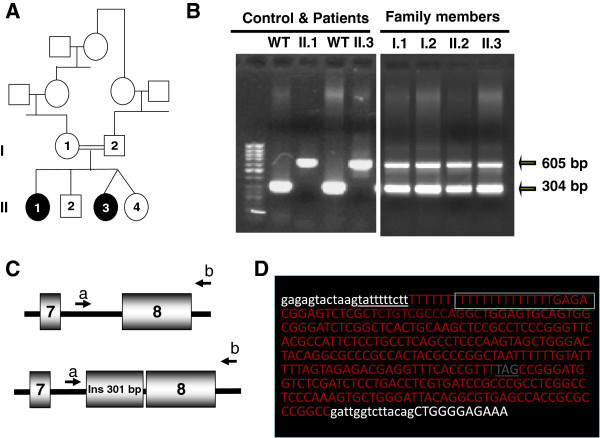
**Panel A: Pedigree of the family #13.** Panel **B**: PCR of exon 8 at agarose gel electrophoresis of siblings #13-II.1 & #13-II.3 and, WT showing, patient #13-II.1 & #13-II.3 were homozygous for 301 bp insertion by having a single band of 605 bp size instead of 304 bp. The other healthy family members were carriers for the insertion, due to band of both 304 and 605 bp size. Panel **C**: Schematic presentation of *CTSK* exon 7 & 8, 301 bp insertion and forward **(a)** and reverse **(b)** primer sites. Panel **D**: Sequencing results from forward primer located at intron 7 and reverse primer from exon 8 of the patients #13-II.1 showing insertion starting from intron 7 after ctt nucleotide and following with poly (T) which belongs to the human Alu of 301 bp in antisense direction. For nucleotides; small letters represents intron 7, white capital letters and red capital letters represent exon 8, inserted segment, respectively. New splice acceptor site within the inserted segment is given in the box and stop codon as gray capital letters.

Agarose gel electrophoresis results in exon 8 and its boundaries showed that patient #13-II.1 and #13-II.3, who were siblings, had novel homozygous insertions and, their healthy parents and healthy siblings had one normal band and one inserted band (Figure 2). The exact localization of the insertion could be determined by forward and reverse sequencing traces and the insertion was detected at intron 7 close to the exon 8. The 301 bp size of the insertion was determined by sequencing. It was shown that the inserted gene fragment is Alu-sequence inserted in reverse orientation. Furthermore, the insertion showed new potential splicing acceptor site at position 38 of the insertion sequence with a consensus value of 89.2%, and, consensus value for formal (wild type) acceptor site was 87.8% on Human Splicing Finder. This new splicing acceptor site most probably is used for alternative splicing. The mRNA for CTPK contains exon 2, 3, 4, 5, 6, 7, insertion and exon 8; however, the insertion sequence introduced stop codon (TAG) after 54 unrelated amino acids in the proceeding sequence, based on the predicted splicing event. When ribosome synthesizes CTPK protein, it stops protein synthesis in the nonsense codon on m-RNA. Thus, CTPK protein contains all exons including part of peptide encoded by insertion sequence except exon 8. However, we could not show mutant expression and proteins with the experiments, since no *CTSK* mRNA expression in the blood was detected in both healthy subject and the patients.

Further analysis showed that patient #14 presented from different center also had the same insertion and the family denied any consanguinity with the first family.

We detected homozygous mutation in the *CTSK* gene in all patients diagnosed as pycnodysostosis by clinical features. Additionally, no other case with clinically suggestive for pycnodysostosis and without any mutation in *CSTK* was detected in our cohort.

### Ancestral backgrounds of families

All detected mutations in our cohort were homozygous and the parents were consanguineous in 12 families, explaining homozygous mutation in all families. Parental consanguinity was denied in two families (patient #6, and patient #9). Further extensive pedigree analysis in these two families revealed that for patient #9, the parents originated from nearby villages. For patient #6, the paternal grandmother of the patient originated from a neighboring village of the mother’s family suggesting that the consanguinity existed in several generations back between the parents of both families.

The patients were presented to three different centers in Istanbul, Ankara and Erzurum, where the families were currently residing.

As detailed in the table, the L7P and R312X mutations were each shared by four and three families respectively, while I249T and the Alu insertion mutations were found in two unrelated families. This observation led us to go back and specifically investigate where ancestors of the families lived before their current city of residence. We were able to detect the villages where the families immigrated to cities for all families. The families of #2 & #3 and, #4 & #5 for L7P, as well as #10 & #11 for R312X and #13 & #14 for Alu insertion mutation used to live in close villages, even these villages remained within the border of different cities. However, families #12 for R312X and #8 & #9 for I249T did not originate from very close regions to other families; however, they are from the same geographical region of the country. So far, the close proximity of common mutations suggests common ancestry. Additional file 4: Figure S3 demonstrates the geographical distribution of all families.

## Discussion

**Figure 3:**
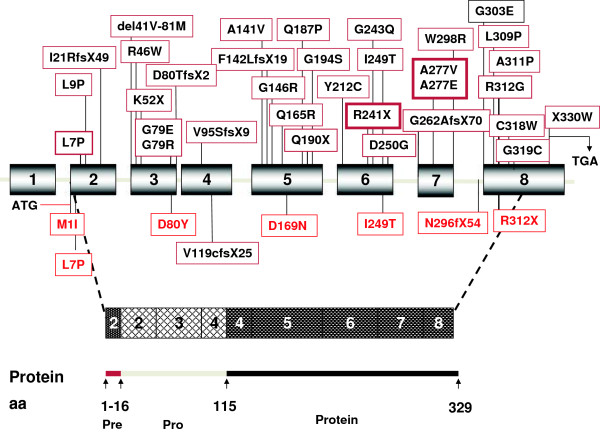
**The schematic presentation of the CTSK gene and protein: The genomic structure of the CTSK gene with 8 exons in the top half and the polypeptide comprising a 15-amino acid preregion, a 99-residue proregion, and a 215-amino acid mature domain in the bottom half.** A total of 33 reported mutations are shown on gene schema on the upper panel, mutation given in thick frames are hot spot mutations, mutation detected herein with red letters and another mutation detected in Turkish families in black letters are shown on the lower panel.

In this study, we identified seven different homozygous *CTSK* mutations in sixteen Turkish children with pycnodysostosis from 14 families. Figure 3 shows previously described mutations in the upper panel and the five novel mutations described in this paper in the lower panel. All mutations detected were homozygous and the parents were consanguineous in twelve families. Two families, who denied consanguinity, were later found to be originating from nearby villages. Four of the described mutations were present in more than one family. This observation suggests the presence of founder mutations in patients originating from geographically close cities in Anatolia. The founder mutations probably pooled for centuries with the help of high consanguineous marriage rate. In fact, the regions where most families originate, namely Northern and Southeastern Anatolia have a higher percentage of consanguineous marriages (up to 40%) well above the average of Turkey (21%) [17]. Furthermore, L7P and I249 mutations, have been described previously from Italian and Spanish patients, respectively, as a part of compound heterozygous mutations [4]. We speculated that the mutated alleles could originate from a common ancestry. Additionally, only one novel mutation (V119cfsX25) has been detected previously in two Turkish families living in nearby villages [18]. This mutation is not detected in our cohort, the reason of this; families in this study come from the North East region of the country while these two families are from the Western part of the country.

In literature, several mutations have been reported in patients with pycnodysostosis. There are 23 different missense mutations (69.70%), three nonsense mutations (9.09%), two frame-shift duplication mutations (6.6%) and two frame-shift deletion mutations (6.6%), two splicing mutations, (6.06%), and one stop codon mutation (3.03%) [6]. Distribution of reported mutations in *CTSK* gene showed that 70% of mutations were found in the mature domain, 24.24% mutation in the proregion and 6.06% mutations in the preregion of *CTSK* gene [6]. In our cohort, 71% of mutations are missense, 14% of mutations are nonsense, while 14% have insertion mutations. The ratio of missense and nonsense mutations in our cohort is similar to those described in the literature; however, the insertion mutation is novel and noteworthy. Localization of mutations in our cohort showed four mutations in the mature domain (57%), which is consistent with literature showing that most mutations are located in the mature domain. We detected two mutations in the predomain and one mutation in the prodomain of CTSK protein (Figure 3).

Intron 7–301 bp insertion mutation (IVS7-14-15insAlu:HSU18392) is the first large insertion mutation described in *CTSK* gene. It was revealed that the insertion was the human Alu element, which is the mobile element in human genome and makes insertion all through the human genome and mostly in intronic region. The *Alu* element consist of about 280 bases in length of main body with a short A-rich region, called as Poly(A) tail [19]. The 3’ end of an *Alu* element has a longer A-rich region that plays a critical role in its amplification mechanism [20]. Here, our insertion segment had 20 bp of the polyadenylation site seen as T, in antisense direction. Alu elements have a tendency to integrate to the AT-rich sites of the genome i.e. 5’-TTAAAA-3’/5’-TTTTAA-3’ [21]. The poly(T) was detected at the downstream of Alu insertion segment at intron 7 of *CSTK* in our case*,* most probably it serves as the insertion site for poly(A) tail for Alu sequence.

*Alu* insertions contribute to diseases by either disrupting a coding region or a splice signal [22,23]. In our case, alternative splicing involving *Alu* elements, known as exonization [24] and premature stop codon after unrelated 54 aa has been occurred.

It is estimated that new *Alu* insertion occurs about one per 20 human births [25], and one in every 1000 insertions cause a new human genetic disease [22]. Alu element insertions are described in many conditions including cancers and polymorphisms related to common diseases in the population [26]. Alu insertion is well described in angiotensin converting enzyme (ACE) polymorphism, and its relation to hypertension, diabetes and metabolic syndrome [27-29]. The Alu insertion in intron 16 of *ACE* leads to decreased enzyme activity with more severe in homozygous states [30]. This condition is very similar to our mutation detected here, since both have decreased enzyme activity.

The R241X and A277V or A277E mutations were previously described as hot spot mutations for *CTSK* gene [6]. However, none of these hot spot mutations have been detected in our cohort.

The remarkable clinical features of patients in our cohort were the atypical fractures and short stature in addition to osteosclerosis, which was present in all patients (Figure 1). Two of the patient did not show any acroosteolysis (#8 and #13.II), although patients who were carrying the same homozygous mutations had acroosteolysis. The absence of acroosteolysis may be misleading in these cases, and typical facial dysmorphic features and family history let us to the diagnosis. However, in a cases series, Pangrazio et al. detected *CTSK* mutations in patients with classical features of osteopetrosis, like blindness, anemia or bicytopenia, splenomegaly and suggestive pedigree of autosomal recessive osteopetrosis, by exome sequencing. Patients in this group had osteosclerosis, but not acroosteolysis, which is a typical and discriminative radiological feature of pycnodysostosis [31]. Furthermore, our patient #8, who did not have acroosteolysis, also had thrombocytopenia and craniosynostosis; however, none of the other patients including patient #13.II had any classical features of osteopetrosis, other than osteosclerosis. We conclude that the absence of acroosteolysis does not exclude the diagnosis of pycnodysostosis, and could be a sign of more severe phenotype with the classical osteopetrotic features.

Additionally, height SDS of the patients are changing from -1.77 to -5.2 SDS in our cohort and, it seems that there is no specific height difference between patients carrying different mutations. Furthermore, height SDS of the patient who carrying the same mutation does not show any trend with age, since better height SDSs were detected in older patients in M1I and R312X group. This is the first paper studying the genotype phenotype correlation, and no genotype phenotype correlation could be found. However, the age of the first bone fracture and probably severity of the fractures could be related to the type of protein defect, since patients with M1I mutations were the youngest patients with fractures in our cohort and, in which translation initiation codon changed and the complete absence of CSTK protein is suggested. The youngest patient with fractures described in literature is 10 months old, in whom mutation analysis was not performed; however, his two sibling with pycnodysostosis died due to the disease suggesting the more severe phenotype and/or genotype in that family [32].

In our cohort, we found that one pycnodysostosis patient had Arnold Chiari malformation and another had craniosynostosis. Craniosynostosis has previously been described in four other cases with pycnodysostosis [32-35]. More recently, Arnold Chiari malformation was reported in the paper of Pangrazio et al. with classical osteopetrosis phenotype of the disease [31]. So far, craniosynostosis seems not to be a rare entity and Arnold Chiari malformation can also be seen in pycnodysostosis.

In conclusion, analyses of the *CTSK* gene in our pycnodysostosis cohort resulted in the description of five novel mutations including one large insertion in the *CTSK* gene, with a possible founder effect. The hot spot mutations previously described (R241X and A277V or A277E) were not detected in this cohort, indeed, L79 and R312X were the most frequent mutation in our cohort of patients with pycnodysostosis.

## Competing interests

The authors declare that they have no competing interests.

## Authors’ contributions

ST, AB, AA participated in the design of the study, writing the manuscript and the discussion of the results. AA and AÇ carried out the molecular genetic studies. ST and AA analyzed the data. YA participated in the discussion of the results. PÖŞK, AB, TG, BÖ, ZA, BH, TA, KB, YA, ST recruited the patients. ST coordinated the study. All authors read and approved the final manuscript.

## Supplementary Material

Additional file 1: Table S1 PCR Primers for *CTSK* gene: The primers were used for the amplifications of specific exons and exon/intron boundaries for exon 2–8. F and R show forward and reverse primers respectively. Click here for file

Additional file 2: Figure S1 The sequencing traces of the patients with *CTSK* mutations, normal sequence shown on upper, mutated sequence shown on lower panel. **A)** M1I Mutation: A normal person has Methionine amino acid residues as translational initiation codon on *CTSK* gene encoded by ATG, G residue change to A to of ATA in the patients. **B)** L7P Mutation, The normal person contains Leucine **(L)** encoded by CTG and the patient has proline **(P)** encoded by CCG created by changing of T residue of CTG codon to C residue. **C)** D80Y Mutation: The control showed GAT codon encoding Aspartic acid **(D)** and the patient has TAC encoding Tyrosine **(Y)** created by changing of G residue of GAT codon to T residue forming TAC codon. **D)** D169N mutation: A normal person has GAT codon at positioned 169 encoding Aspartic acids **(D)** and G residue of GAT was substituted with A residue to make AAT codon encoding asparagines **(N)**. **E)** I249T mutation: Isoleucine **(I)** encoded by ATT found at healthy people and I249T mutation was created by conversion of the first T residue of ATT to C to make ACT encoding Threonine **(T)**. **F)** R312X: The change of C residue of CGA encoding Arginine **(R)** to T resulted in TGA resulted in a stop codon. *Shown in reverse sequence. Click here for file

Additional file 3: Figure S2 The sequence traces of the mutations I249T and D169N. The parents are heterozygous for the mutation and the patients are homozygous for ATT to ACT and GAT to AAT nucleotide changes. Click here for file

Additional file 4: Figure S3 The mutation map of *CTSK* gene in Turkey showing that the same mutation originates from the neighboring geographical regions: Mutations are given according to the latitude of the country where the families originally located at upper panel. Click here for file
