# Supplementary material for: Cathepsin K analysis in a pycnodysostosis cohort: demographic, genotypic and phenotypic features
Source: Orphanet J Rare Dis. 2014 Apr 26;9:60. doi: 10.1186/1750-1172-9-60 (PMC4022088; doi:10.1186/1750-1172-9-60)
Supplement: Additional file 1: Table S1 — PCR Primers for CTSK gene: The primers were used for the amplifications of specific exons and exon/intron boundaries for exon 2–8. F and R show forward and reverse primers respectively. [file 1750-1172-9-60-S1.pdf]

**1-CATK-E234F**	5’-TTCTCTTCCTCATAAAGCCTAG-3’
**2- CATK-E234R**	5’-TCACCTCAAGAACAAAGCAGCAG-3’
**3- CATK-E5F**	5’-TCAGGCAGGTAGAGAGAAGATATAG-3’
**4- CATK-E5R**	5’-AAGCAGGATAGGATAACAGA-3’
**5- CATK-E67F**	5’-ACTGCTGCCTCTGTTAGTTCACTG-3’
**6- CATK-E67R**	5’-AAGATAGAAGTGAGAACTCTGAG-3’
**7- CATK-E8F**	5’-ATCAGTACCTCGCACAATACTC-3’
**8- CATK-E8R**	5’-ATCTCAGTATCACCACATCTGC-3’
