# Supplementary figures and images for: Cathepsin K analysis in a pycnodysostosis cohort: demographic, genotypic and phenotypic features
Source: Orphanet J Rare Dis. 2014 Apr 26;9:60. doi: 10.1186/1750-1172-9-60 (PMC4022088; doi:10.1186/1750-1172-9-60)

## Slide 1
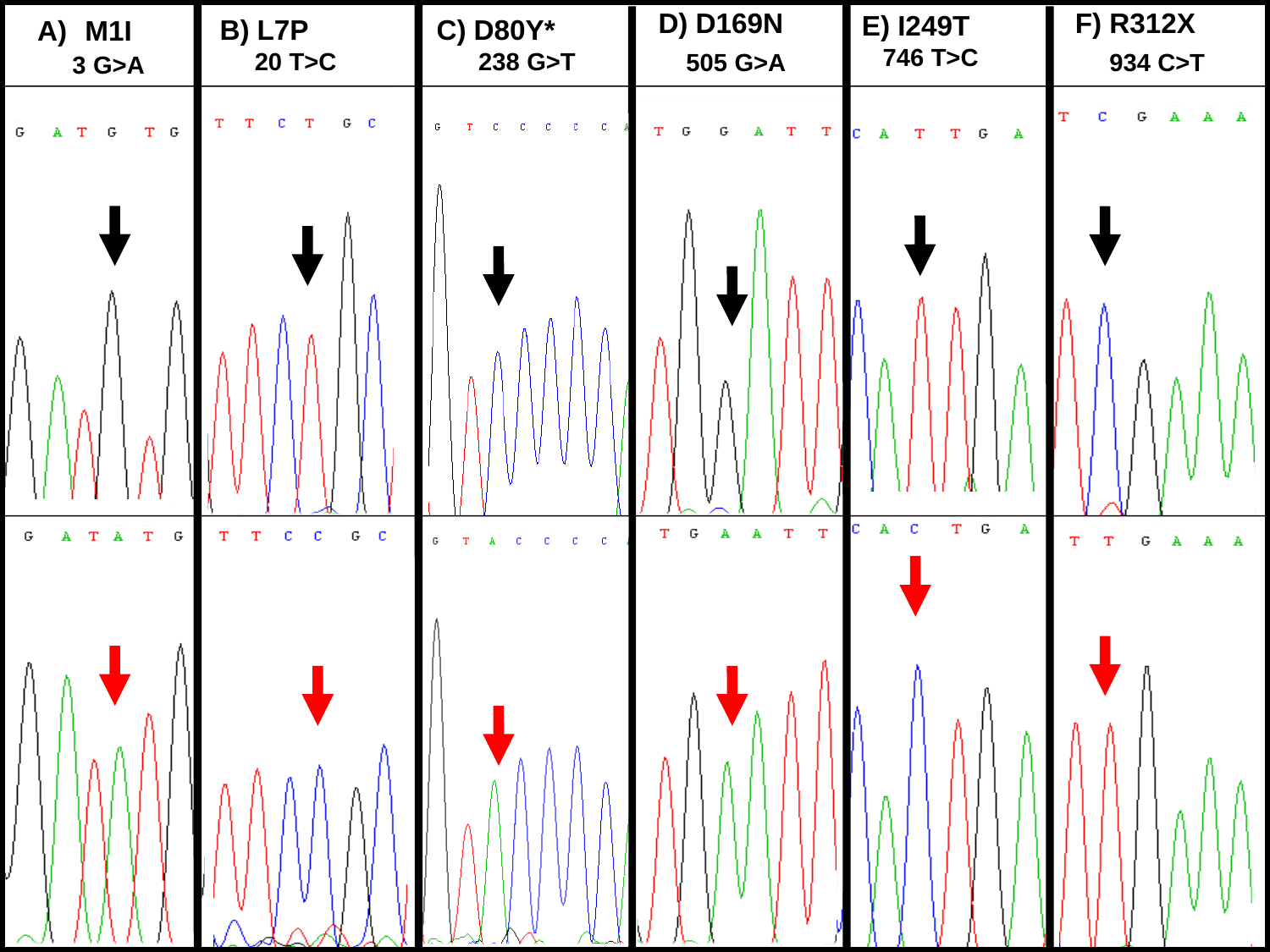

D) D169N
 505 G>A
 F) R312X
 934 C>T
E) I249T
 746 T>C
B) L7P
 20 T>C
C) D80Y*
 238 G>T
M1I
 3 G>A

Supplement: Additional file 2: Figure S1 — The sequencing traces of the patients with CTSK mutations, normal sequence shown on upper, mutated sequence shown on lower panel. A) M1I Mutation: A normal person has Methionine amino acid residues as translational initiation codon on CTSK gene encoded by ATG, G residue change to A to of ATA in the patients. B) L7P Mutation, The normal person contains Leucine (L) encoded by CTG and the patient has proline (P) encoded by CCG created by changing of T residue of CTG codon to C residue. C) D80Y Mutation: The control showed GAT codon encoding Aspartic acid (D) and the patient has TAC encoding Tyrosine (Y) created by changing of G residue of GAT codon to T residue forming TAC codon. D) D169N mutation: A normal person has GAT codon at positioned 169 encoding Aspartic acids (D) and G residue of GAT was substituted with A residue to make AAT codon encoding asparagines (N). E) I249T mutation: Isoleucine (I) encoded by ATT found at healthy people and I249T mutation was created by conversion of the first T residue of ATT to C to make ACT encoding Threonine (T). F) R312X: The change of C residue of CGA encoding Arginine (R) to T resulted in TGA resulted in a stop codon. *Shown in reverse sequence. [file 1750-1172-9-60-S2.pptx]

## Slide 1
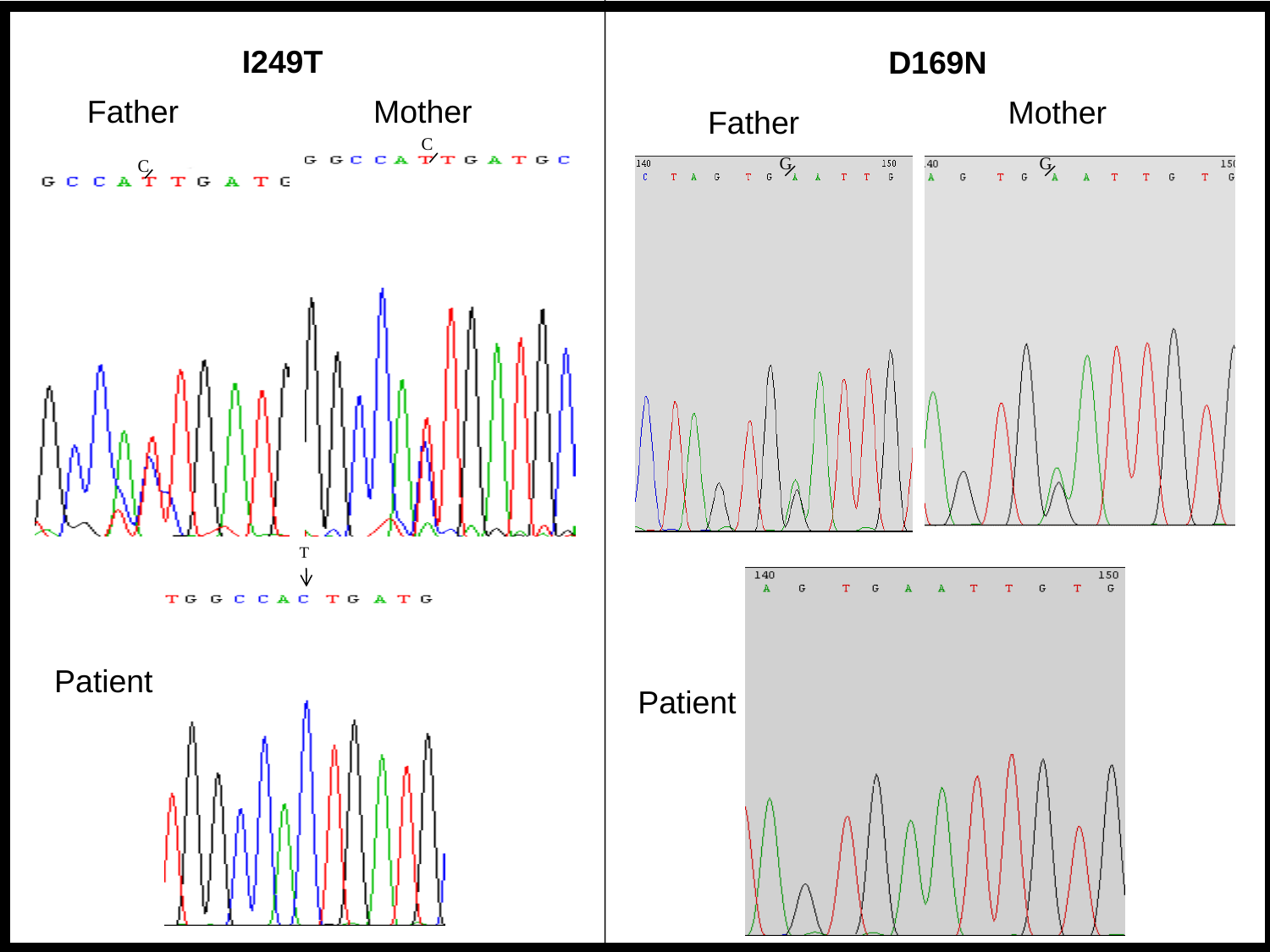

I249T
D169N
Father Mother
Mother
Father
C
G
G
C
T
Patient
Patient

Supplement: Additional file 3: Figure S2 — The sequence traces of the mutations I249T and D169N. The parents are heterozygous for the mutation and the patients are homozygous for ATT to ACT and GAT to AAT nucleotide changes. [file 1750-1172-9-60-S3.pptx]

## Slide 1
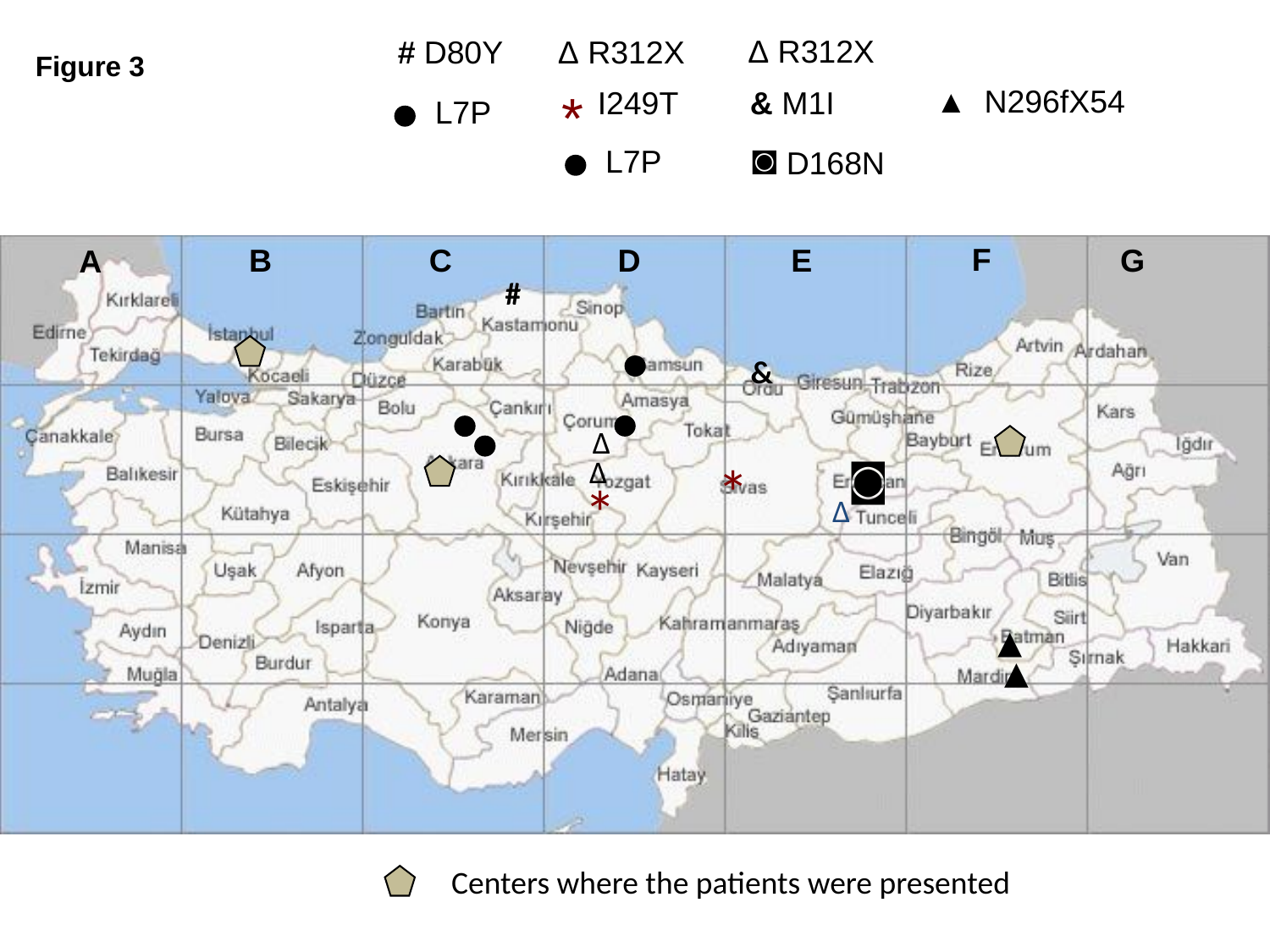

Δ R312X
# D80Y
Δ R312X
Figure 3
▲ N296fX54
*
I249T
& M1I
L7P
◙ D168N
L7P
F
B
C
D
E
G
A
#
&
Δ
Δ
*
◙
*
Δ
▲
▲
Centers where the patients were presented

Supplement: Additional file 4: Figure S3 — The mutation map of CTSK gene in Turkey showing that the same mutation originates from the neighboring geographical regions: Mutations are given according to the latitude of the country where the families originally located at upper panel. [file 1750-1172-9-60-S4.pptx]
